# Primary extragenital endometrial stromal sarcoma of the lung: first reported case and review of literature

**DOI:** 10.1186/s13000-017-0627-2

**Published:** 2017-05-02

**Authors:** Lara Alessandrini, Francesco Sopracordevole, Giulio Bertola, Simona Scalone, Martina Urbani, Gianmaria Miolo, Tiziana Perin, Fabrizio Italia, Vincenzo Canzonieri

**Affiliations:** 1 Pathology, IRCCS-National Cancer Institute, Via F. Gallini 2, 33081 Aviano, Italy; 2 Gynecological Oncology Unit, IRCCS-National Cancer Institute, Aviano, Italy; 3 Surgical Oncology, IRCCS-National Cancer Institute, Aviano, Italy; 4 Department of Medical Oncology, IRCCS-National Cancer Institute, Aviano, Italy; 5 Department of Radiology, IRCCS-National Cancer Institute, Aviano, Italy; 6 Oncopath Lab, Floridia, Siracusa Italy

**Keywords:** Extragenital endometrial stromal sarcoma, Lung, Immunohistochemistry, Case report

## Abstract

**Background:**

Endometrial stromal sarcomas arising in extrauterine and extraovarian sites, in the absence of a primary uterine lesion are quite rare, especially in the absence of endometriosis. They usually present as an abdominal or pelvic mass lesion.

**Case presentation:**

In 2007, a 45-year-old woman underwent total hysterectomy for in situ squamous cell carcinoma of the cervix. In 2014, an upper left pulmonary lobectomy was performed for a mass, which was provisionally diagnosed as primary carcinosarcoma of the lung. A second histological revision of the lung surgical specimen was performed in the Pathology Unit of our Institute. After extensive immunohistochemical analyses, the preferred diagnosis was spindle-cell sarcoma, consistent with high-grade extragenital endometrial stromal sarcoma (EESS). A review of all slides of the hysterectomy specimen confirms the original diagnosis: no evidence of stromal tumor was found. Afterwards, the patient developed multiple and metachronous pulmonary lesions and a scapular soft tissue mass, which showed the same morphophenotypic features of the first lung mass. The patient was treated with antiblastic therapy, surgical resection and radioablation, when appropriate. To date, the patient has no signs or symptoms.

**Conclusions:**

The authors present the first case of primary EESS arising in the lung with no association with endometriosis published to date. Detailed clinical history and follow-up are also described. Moreover, extensive literature review is reported, along with differential diagnoses, immunohistochemical and molecular findings, pathogenetic hypotheses and treatment options. The knowledge of EESS potential extrauterine location and of its peculiar morphophenotypic aspects are required for a correct diagnosis, and for choosing the most suitable treatment.

## Background

Endometrial stromal sarcoma (ESS) is an uncommon mesenchymal tumor of the uterus, which accounts for less than 1% of all uterine malignancies but it is the second most common uterine malignant mesenchymal tumor [1]. The latest World Health Organization Classification [1], categorizes ESS into low-grade ESS (LGESS), high-grade ESS (HGESS) and undifferentiated endometrial sarcoma (UES). LGESS are typically composed of cytologically bland fusiform cells resembling stromal cells of proliferative-phase endometrium, intermingled with numerous small plexiform arterioles, permeating the myometrium as well as the intramyometrial or parametrial vessels. The mitotic rate is usually lower than 5 mitoses/10HPF. They show an indolent course: recurrences and metastases are rare and occur after long periods of time [1, 2]. In contrast, both HGESS and UES have a malignant behavior. They often exhibits myometrial invasion, hemorrhage and necrosis, as well as marked nuclear pleomorphism with round cell morphology (and a minor component part of low-grade spindle cell morphology) and high mitotic activity with 10 mitoses/10HPF or higher. Patients are more likely to develop recurrences and die from disease. The most important morphologic feature that distinguishes ESS from UES is that the latter lacks overt resemblance to proliferative endometrial stroma.

Endometrial stromal sarcomas arising as primary tumors in extrauterine and extraovarian (i.e., extragenital endometrial stromal sarcomas) sites are quite rare, and reported in the English medical literature as small series or case reports (Table 1) [3–29].

**Table 1:** Clinical characteristics of EESS cases presented in Literature

Ref	Site	N° of cases	Age (Range)	N° of cases with history of endometriosis	N° of cases with history of gynecologic surgery	N° of cases with concurrent endometriosis	Treatment (N° of pts)	Follow-up: time (range) and outcome (N° of pts)
3,6,17, 21,26	Pelvis	6	34–50	2	1	4	resection + radioTX (1) resection (3) NA (2)	10 mo-1y; DOD (1), NED (2), NA(3)
6,13, 15, 17,18	Omentum, abdomen, retro-peritoneum, mesentery	5	46–71	1	1	3	resection + radioTX (1) resection (4)	11 mo-4y; NED (3), NA(1); recurrence (1)
5,10,11, 12,14, 19,20, 21,23, 27, 28	Small bowel, colon, rectum	11	38–80	3	5	6	resection + radioTX (1) resection + chemoTX (3) resection (6) NA (1)	4 mo-4y; DOD (1), NED (6), NA(2); recurrence (2)
22	omentum, mesentery, colon, liver, bladder, abdominal nodes, pelvis, vagina, aryepiglottic fold		38 cases EESS; age:27–81 (range)	NA	NA	14/38 cases	resection + chemoTX +/− radioTX	NED (12/38 cases, range : 15–174 mo) DOD (8/38 cases; range 36–336 mo); AWD (13/38 cases; range:6-145mo)
25	stomach		37–54	NA	1	none	resection	NA
13	Liver	1	31	1	1	1	resection	NED; 4 y
4,8,9, 16,17, 24	vagina	6	32–45	none	1	none	resection + radioTX (2) resection + chemoTX (1) resection (3)	18 mo-38 mo; NED (5), NA (1)
7,29	vulva	2	34–50	none	2	1	Resection (2)	28 mo-6 y NED (2)

*TX* therapy, *NED* no evidence of disease, *DOD* dead of disease, *NA* not available, *mo* months, *y* year/s, *AWED* alive with disease

The clinical-pathological features of primary EESS have not been widely investigated yet. To the authors’ knowledge, this is the first case reported of EESS arising in the upper left lobe of the lung in a 45-year-old woman without associated endometriosis and in the absence of a primary genital ESS. An extensive review of literature is also reported, along with differential diagnoses, immunohistochemical and molecular findings, pathogenetic hypotheses and treatment options.

## Case presentation

### Clinical history

In 2007, a 38-year-old female was hospitalized for an ulcerated uterine cervical lesion which, after cervical biopsies, showed an in situ squamous cell carcinoma. Subsequently, an abdominal CT scan revealed an enlarged, dishomogeneous uterus, with hypodense mass of 5 cm adjacent to the posterior wall of the bladder. Two months later, the patient underwent total abdominal hysterectomy with bilateral salpingectomy and pelvic lymphadenectomy. In the intraoperative phase, the uterus was found to be mobile and ovaries were normal. Neither enlarged lymph nodes nor peritoneal lesions or ascites were noted.

On histological examination of the surgical specimen, a well-differentiated squamous cell carcinoma in situ of the cervix, measuring 4 mm in maximum diameter, with basaloid morphology, was identified. Three additional leiomyomata of the uterine corpus, besides the larger one already identified on CT scan, were also microscopically found.

**Fig. 1:**
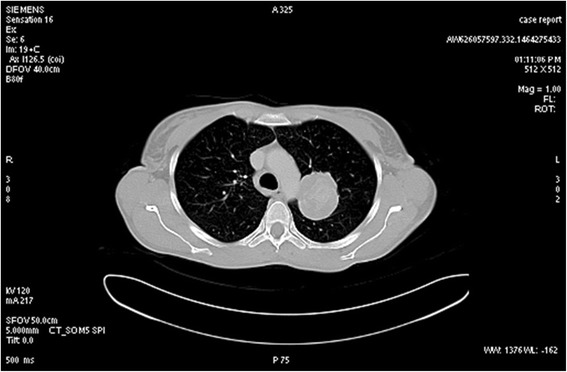
Thoracic CT scan showing a mass (50 mm of maximum diameter) in the upper left lobe

In 2014, the patient presented to medical observation with a pulmonary mass (Fig. 1). Upper left lobectomy was performed. The final pathologic diagnosis was carcinosarcoma of the lung.

A second histological revision of the lung surgical specimen was performed in March 2014 in the Pathology Unit of our Institute. The preferred diagnosis, after extensive immunohistochemical analyses, was spindle-cell sarcoma, consistent with high grade EESS.

Post-operative staging with thoracic, abdominal and pelvic CT scan did no have any abnormal findings and a follow-up was proposed. After five months, a CT scan was performed: it revealed a left pulmonary lesion with a maximum diameter of 18 mm. Chemotherapy treatment with carboplatin AUC 5 and paclitaxel 175 mg/mq every three weeks was started. After three courses of antiblastic therapy, the lesion in the left lung appeared to be increased in volume (27 mm of diameter) on CT scan. After multidisciplinary discussion, a surgical intervention (atypical segmentectomy of lower lobe of the left lung) was performed.

Almost three months after surgical intervention, a new pulmonary lesion located in the right lung was detected by CT scan. The lesion was subsequently treated with radiofrequency ablation. As a consequence of the procedure, right pleural effusion after 24 h and after five days by the appearance of right pneumothorax after five days appeared. Shortly after tube insertion, additional radiographs were taken: they showeda rapid decrease in the size of the pneumothorax. The thoracic drainage was removed and patient was discharged from the hospital.

After one month, a CT scan was repeated. The scan showed surgical and radiofrequency ablation effects and a new pulmonary lesion of uncertain nature, measuring 4–5 mm of maximum diameter. Hormonal therapy with progestin (acetate medroxyprogesterone 1 gr daily) was prescribed.

No radiological signs of progression were noted until December 2015, when new lung lesions located in the lower lobe of the right lung and upper lobe of the left lung were detected on CT scan.

In January 2016, the patient referred the appearance of a soft tissue mass, rapidly enlarging, located in the left periscapular region. A tru-cut biopsy of the soft tissue lesion was performed: histological examination revealed a neoplastic lesion with spindle cell morphology, reminiscent of the upper left lobe EESS.

A second line polychemotherapy with epirubicin and ifosfamide was started. After three courses of chemotherapy, radiological evaluation through CT scan revealed a decrease of the diameter of the right lung lesion and the disappearance of the lesion in the left lung. At the follow-up visit, the patient was in good conditions well, with no clinical signs or symptoms of the disease and no pathological findings on CT scan; routine laboratory tests were normal.

Signed written consent was obtained from the patient for this case report.

## Methods

Two and half-micron sections from formalin-fixed paraffin embedded tissue of the resected lobe and of the hysterectomy specimen were cut and immunohistochemical analysis was performed through an automated system (Benchmark-XT, Ventana, Tucson, AZ, US). The following primary antibodies were used:

CD117 (pathway c-kit, clone 9.7, pre-diluted; Ventana, Tucson, AZ, US), Estrogen receptor (monoclonal antibody, clone SP1, prediluted, Ventana), Progesteron receptor (monoclonal antibody, clone 1E2, prediluted, Ventana), Ki67 (monoclonal antibody, clone 30.9, prediluted, Ventana), CD10 (monoclonal antibody, clone Sp67, prediluted, Ventana), CD34 (monoclonal antibody, clone QBEND/10; 1:400 dilution; Neomarkers, Freemont, CA, USA), CD31 (monoclonal antibody, clone JC70, Prediluted, Cell Marque, Rocklin, CA, US), Pankeratin (CkAE1/AE3/pCk26, pre-diluted, Ventana), MNF116 (monoclonal antibody, clone MNF116, prediluted, Diagnostic Biosystem, Pleasanton, CA, US), smooth muscle actin (SMA) (monoclonal antibody, clone 1A4, 1:100 dilution; DAKO, Glostrup, Denmark/Carpinteria, CA, US), H-Caldesmon (monoclonal antibody, clone E89, prediluted, Ventana), S100 (polyclonal antibody, 1:400 dilution; DAKO), MDM2 (monoclonal antibody, clone IF2, 1:100 dilution, Calbiochem, Merk, Darmstardt, Germany), D2-40 (monoclonal antibody, clone podoplanin, prediluted, Cell Marque), CD99 (monoclonal antibody, clone o13, prediluted, Ventana), Myogenin (monoclonal antibody, clone FD5, 1:50 dilution, Cell Marque), Myoglobin (polyclonal, prediluted, Ventana), Vimentin (monoclonal antibody, clone V9, prediluted, Ventana), Desmin (monoclonal antibody, clone De-R-11, prediluted, Ventana), TTF1 (monoclonal antibody, clone 8G9G3/1, prediluted, Ventana).

The color was developed with 3.3′-diaminobenzidine (DAB), and the slides were counterstained with Meyer’s hematoxylin. Appropriate positive and negative controls were run concurrently.

### Morphological findings

**Fig. 2:**
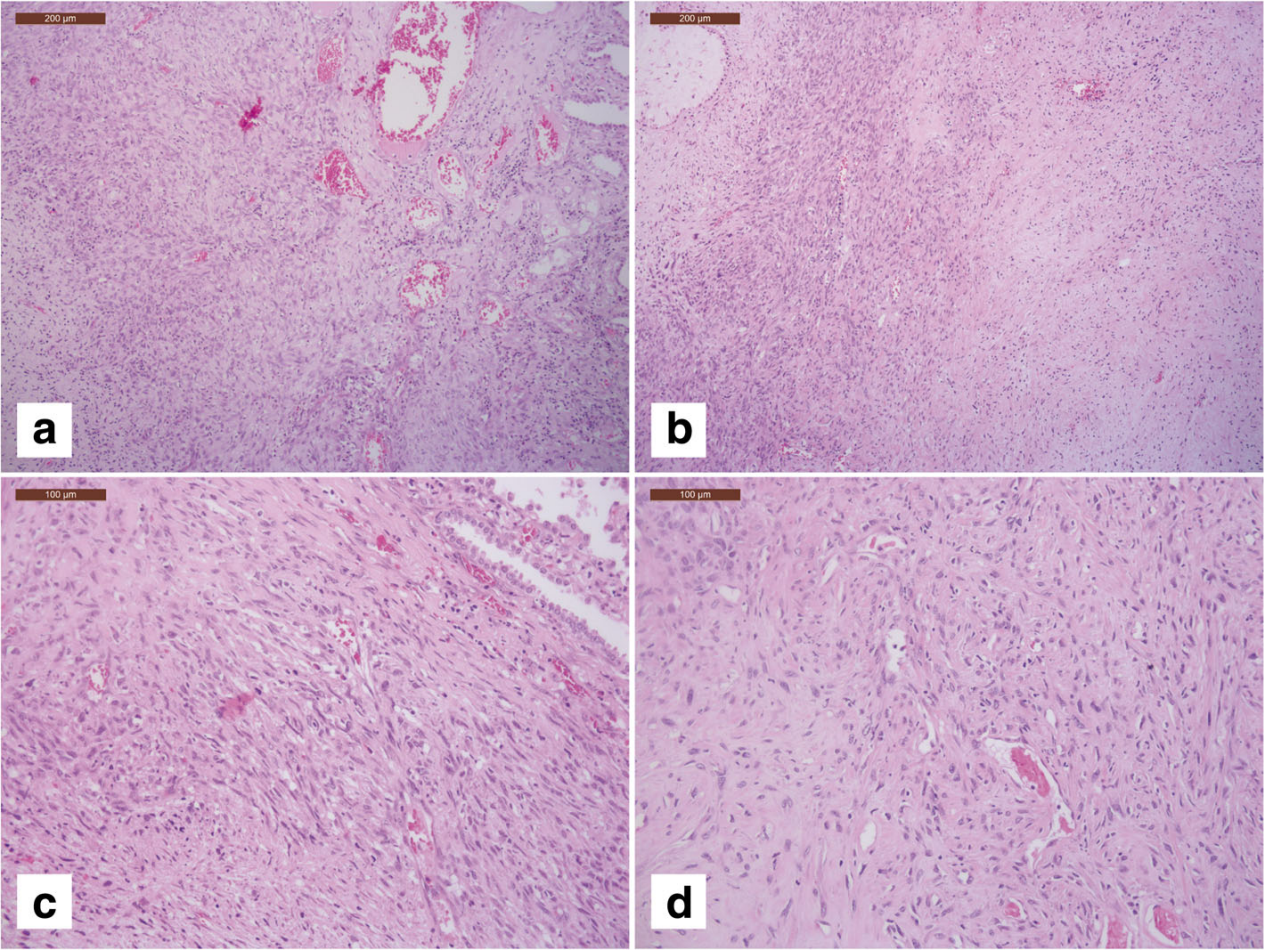
**a**, **b** Panoramic view of the pulmonary lesion showing uniform spindle cells arranged in a fascicular pattern; alveolar epithelium is focally entrapped by the neoplastic cells (H&E, 100×); **c**, **d** some small thinwalled blood vessels resembling spiral arterioles of late secretory endometrium were unevenly distributed among the stroma (H&E, 100×); **b**, **d**, neoplastic cells show moderate atypia and foci of hyaline-type necrosis, in a background of fibromyxoid stroma); **d**, the tumor cells had oval to plump spindle-shaped nuclei with finely granular chromatins, inconspicuous nucleoli, and amphophilic cytoplasms (H&E, 200×)

The histological examination of hematoxylin and eosin stained slides of the pulmonary lesion showed uniform spindle cells organized in a diffuse pattern (Fig. 2a, b, c), with mild atypia and foci of necrosis (Fig. 2d), in a background of fibromyxoid stroma (Fig. 2d). The tumor cells had oval to round nuclei with inconspicuous nucleoli, and eosinophilic cytoplasms. Some small-sized thick-walled vessels were unevenly distributed among the stroma (Fig. 2a, d). Mitotic rate was high, ranging up to 20 mitoses per 10 high-power fields. Alveolar epithelium was focally entrapped by the neoplastic cells (Fig. 2a, c). Despite a thorough examination, endometriotic spots were not identified in the tumor or in adjacent non-neoplastic tissue.

A review of all slides from the hysterectomy specimen, which included the large lesion identified on CT scan plus three additional microscopically found leiomyomas, confirmed the original diagnosis of squamous carcinoma in situ of the cervix with leiomyomas of the uterine wall. No evidence of ESS or endometrial stromal nodules was found.

### Immunophenotipic findings

**Fig. 3:**
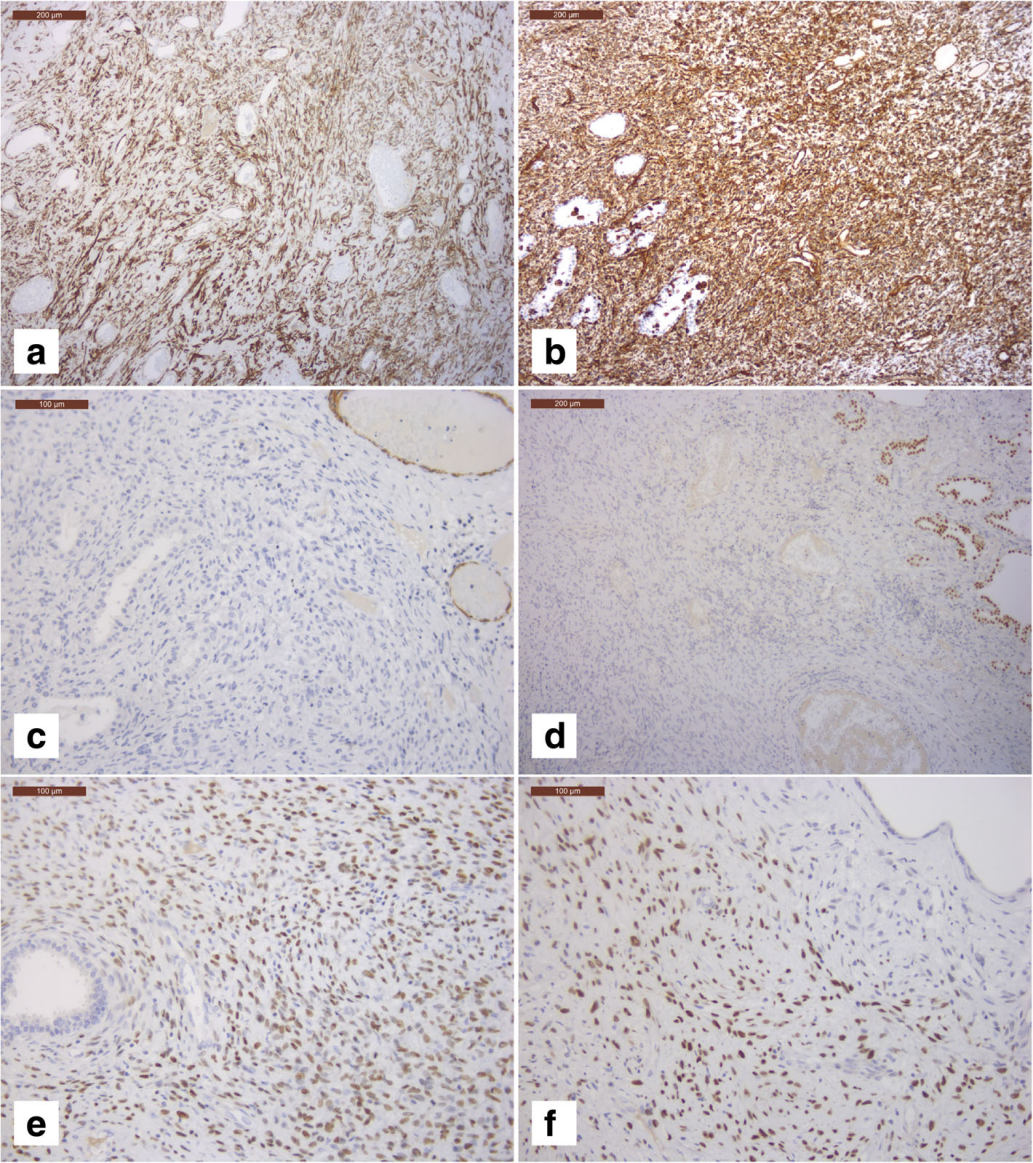
Neoplastic cells showed patchy and intense immunoreactivity for CD10 (**a**, 100×), diffuse vimentin staining (**b**, 100×), negative staining for H-Caldesmon (positive control staining in small vessels;upper left) (**c**, 100×), negative staining for TTF-1 (positive nuclear staining in the entrapped epithelial alveolar elements; right) (**d**, 100×), moderately diffuse nuclear staining for estrogen receptor (**e**, 200×), moderately diffuse nuclear staining for progesterone receptor (**f**, 200×)

On immunohistochemical staining, the tumor cells showed patchy and intense immunoreactivity for CD10 (Fig. 3a), estrogen receptor (Fig. 3e), and progesterone receptor (Fig. 3f), focal staining for CD99, and diffuse vimentin staining (Fig. 3b). Tumor cells also showed negative results for CD117, S-100 protein, and CD34, CD31, D2-40, MDM2, pan-keratin, H-caldesmon (positive in small vessels) (Fig. 3c), MNF116 (positive in the entrapped epithelial alveolar elements), TTF-1 (positive in the entrapped epithelial alveolar elements) (Fig. 3d), myogenin and myoglobin. Ki67 percentage of positive nuclei ranged from 20 to 40%. Limited smooth muscle differentiation was occasionally present and was focally positive for smooth muscle actin and desmin. The preferred diagnosis was EESS with high grade areas.

**Fig. 4:**
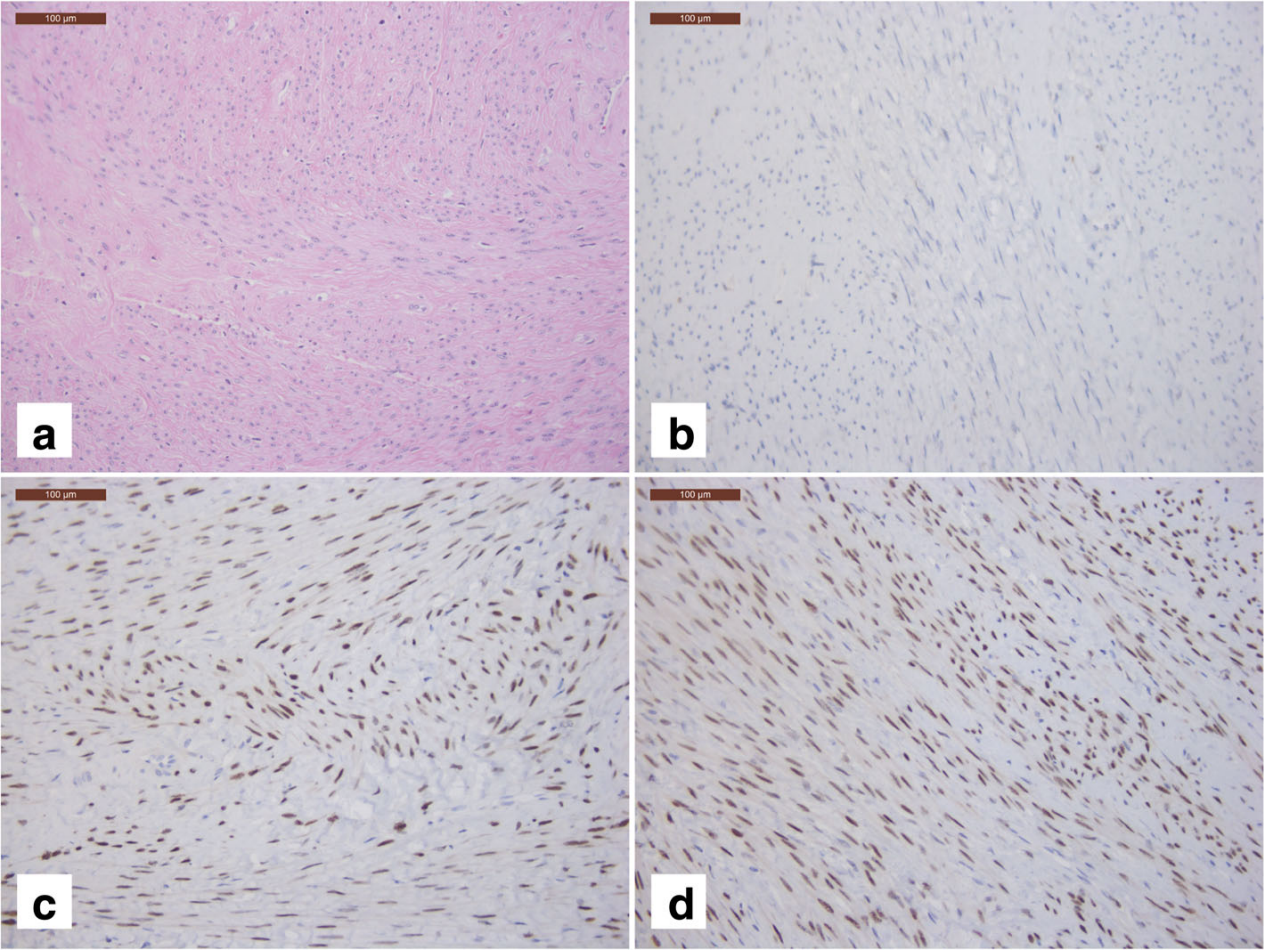
Leiomyoma with classic morphology from previous hysterectomy specimen, showing intersecting fascicles of cigarshaped, spindle cells with eosinophilic cytoplasm (**a**, 200×); CD10 negative immunostaining (**b**, 200×); positive nuclear staining for estrogen receptor (**c**, 200×), positive nuclear staining for progesterone receptor (**d**, 200×)

CD10 was completely negative (Fig. 4b) whereas estrogen and progesterone receptors (Fig. 4c, d, respectively) were positive, as expected, in leiomyomas from the previous hysterectomy specimen.

The periscapular soft tissue recurrence showed the same histological and immunohistochemical findings, except for the absence of staining for CD10, which displayed a patchy staining on the lung specimen; therefore, its absence on a tru-cut biopsy of periscapular soft tissues could be due to sampling from a non-staining area.

## Discussion

Endometrial stromal sarcoma is a malignant tumor closely resembling stromal cells of proliferative-phase endometrial stromal cells. It is commonly associated with a delicate network of arterioles. Even when displaying classical histological features, an unusual site of occurrence may make the diagnosis challenging. Therefore, despite its rarity EESS should always be taken into consideration in the differential diagnosis for a woman with a thoracic or abdominal tumor with uniform cytologically bland cells resembling normal endometrial stroma. On the other hand, in the gastrointestinal tract and extragenital sites, in the absence of endometriosis EESSs may be mistaken for other mesenchymal more common neoplasms [20], such as cellular leiomyoma and low-grade leiomyosarcoma, solitary fibrous tumor and gastrointestinal stromal tumor (Table 2). In such cases, a large panel of immunohistochemical markers could be of help in establishing the diagnosis (Table 2). Regardless of the site of presentation, also peculiar histological features may be challenging for diagnosis, when noted in EESS [22]. When tubules-like structures are identified within spindle cell proliferations, also endometriosis and adenosarcoma are additional diagnoses that should be considered. On the other hand, EESS also can have glandular differentiation. In this case periglandular stromal condensation around benign glands and polypoid fronds composed of cellular stroma imparting a leaf-like appearance are morphological features more characteristic of adenosarcoma.

**Table 2:** Morphophenotypic features of the most common mesenchymal spindle cell neoplasms in the differential diagnosis of EESS

Mesenchymal Neoplasm	Morphological features	Immunohistochemical markers
Cellular leiomyoma/low-grade leiomyosarcoma	characteristic intersecting fascicles of smooth muscle cigarshaped spindle cells with eosinophilic cytoplasm and perinuclear vacuolation, showing a smooth, pushing margin and large, irregular, thick-walled blood vessels	muscle-specific actin +, smooth muscle actin +, desmin +, ER +, PgR+, CD10 −/+, H-caldesmon +
Gastrointestinal stromal tumor	spindle cells with long tapering nuclei and abundant clear or eosinophilic cytoplasm arranged in fascicles or sheets; characteristically well circumscribed with pushing borders	CD34 +, DOG-1 +, CD117 +, CD10 -, ER -, PgR-; muscle-specific actin −/+, smooth muscle actin −/+, desmin −/+,
Solitary fibrous tumor	bland spindle cells haphazardly arranged in a dense collagenous matrix but lacks the peculiar characteristic vasculature of the ESS	CD34 +, CD10 -, ER -, PgR-
Monophasic synovial sarcoma	most commonly involves the soft tissues of the extremities;	CD99 +, EMA +, CD10 -, ER -, PgR-
ESS	monomorphous plump spindle cells forming short regular fascicles, evenly distributed arterioles and infiltrative border	muscle-specific actin −/+, smooth muscle actin −/+, desmin −/+, ER +, PgR+, CD10 +, H-caldesmon -

- negativity, −/+ focal positivity, + diffuse positivity, *ER* estrogen receptor, *PgR* Progesteron receptor, *EMA* epithelial membrane antigen

In the largest group of EESS described to date, one case arising in the larynx was initially diagnosed as monophasic synovial sarcoma [22] which is not usually included into the differential diagnoses of EESS as it frequently arises within the soft tissues of the limbs. Immunohistochemistry can once again be helpful (Table 2). Finally, the long time from primary genital ESS onset and its recurrence and/or metastasis, requires a review of the clinical history of the patient and a second look at the slides from a previous hysterectomy specimen to rule out the possibility of an overlooked uterine ESS [2]. In our patient the hysterectomy specimen was extensively sampled and a review of all slides confirmed the original diagnosis.

Several hypotheses are invoked to explain the pathogenesis of EESS. Since in many cases (Table 1) endometriosis was found to be adjacent to EESS, it could be speculated that primary EESS arises from an ectopic focus of endometrial stroma. However, our case and few others, (listed in Table 1), indicate that the absence of associated endometriosis does not preclude primary EESS at that site. In these cases, either EESS may arise from an underlying endometriosis hidden by sarcomatous overgrowth [22] or it might derive *de novo* from the peritoneal/pleural (in our case) surface or the coelomic or subcoelomic multipotential epithelium [30, 31].

Recent molecular studies showed that ESS can be genetically heterogeneous. The most common alteration carried by ESS is the t (7;17) (p15;q21) translocation, which results in JAZF1–SUZ12 gene fusion (also known as JJAZ1) [32]. However, such rearrangements rarely occur in ESS displaying different morphological features e.g., myxoid, epithelioid, fibrous, smooth muscle, and sex-cord histologic variants: thisreveal that other unknown molecular changes may be involved in the disease phenotypic presentation [32]. Other gene fusionsconcerning PHF1 and YWHAE genes, are much less frequent and are likely to be related with specific clinicopathological features [33]. Although molecular testing for the t (7;17) (p15;q21) and associated gene fusion may be useful for confirming primary extrauterine endometrial stromal sarcoma, the low prevalence of the genetic aberration in this subset of patients limits the clinical utility of the analysis [21].

The behavior of EESS, compared to genital ESS, seems to be controversial. Although results are conflicting, it seems that the only feature associated with a worse prognosis is the presence of morphological features suggestive of dedifferentiation [22].

Given the rarity of these tumors, evidence-based data are not available to help guide treatment decisions. Surgery is generally regarded as the cornerstone of treatment for ESS. Complete cytoreductive surgery is recommended, especially in patients for whom this surgery could result in being residual-disease-free. Tumor-free margins are important in prognosis [34]. Extragenital ESS may have a high tendency for dissemination and metastasis in the omentum, mesentery, and abdominal or pelvic wall [20, 22]. Despite this, most patients with extrauterine ESS had prolonged disease-free intervals with late recurrences [20, 22]. The value of adjuvant therapy is controversial with no prospective studies showing a survival advantage associated with the use of chemotherapy or radiotherapy [34]. Nevertheless, adjuvant therapy is still considered in patients with metastatic or recurrent ESS. Radiotherapy may also have a palliative role [34].

## Conclusions

In summary, we present in this study the first EESS of the lung reported to date. EESS is an uncommon tumor arising in women of any age, usually presents as a mass lesion in the abdomen or pelvic cavity. The knowledge of its potential extrauterine location and of peculiar morphophenotypic aspects are required for a correct diagnosis. Surgical resection is the most frequent treatment option, eventually followed by hormonal-therapy. EESS does not seem to have an aggressive behavior, and is likely to recur late after the initial treatment: therefore, a long-term clinical follow-up should be programmed.

## Abbreviations

CT: Computed tomography
ESS: Endometrial stromal sarcoma
HGESS: High-grade endometrial stromal sarcoma
H&E: Hematoxylin-eosin
LGESS: Low-grade endometrial stromal sarcoma
UESS: Undifferentiated endometrial stromal sarcoma
